# Supplementary material for: Multiplexed Digital PCR Reference Gene Measurement for Genomic and Cell-Free DNA Analysis
Source: Cells. 2025 Oct 3;14(19):1544. doi: 10.3390/cells14191544 (PMC12523864; doi:10.3390/cells14191544)
Supplement: Supplementary file 1 [file cells-14-01544-s001.zip › cells-3837179-supplementary.pdf]

Multiplexed Reference Gene Measurement for genomic and cell-free DNA analysis

Dilek Yener [1,2,*], Eloise J. Busby [1], Jo Vandesompele [3], Gertjan Wils [3], Susan D. Richman [2,4], Henry M. Wood [2,4], Jim F. Huggett [1,5], Carole A. Foy [1] and Alison S. Devonshire [1,*]

1 National Measurement Laboratory, LGC, Guildford, GU2 7XY, UK; jim.huggett@lgcgroup.com (J.F.H.)

2 Leeds Institute of Medical Research at St James's, University of Leeds, LS9 7TF, UK

3 pxlence, 9000 Ghent, Belgium; jo.vandesompele@pxlence.com (J.V.); gertjan.wils@pxlence.com (G.W.)

4 NIHR Leeds Biomedical Research Centre, Leeds, LS7 4SA, UK

5 Faculty of Health & Medical Sciences, University of Surrey, Guildford, GU2 7XH, UK

* Correspondence: dilek.yener@lgcgroup.com (D.Y.); alison.devonshire@lgcgroup.com (A.S.D)

Section 1: Supplementary Methods 3
Table S1: Restriction enzyme digestion protocol 3
Table S2: gBlock™ information 3
Table S3: Hydrolysis Assay Oligonucleotide information 4
Table S4: Pxlence Rainbow™ Assay Oligonucleotide information 5
Table S5: Assay preparation 6
Table S6: Reaction Setup 6
Table S7: Thermal cycling conditions 7
Table S8: Imaging Parameters 7
Section 2: Supplementary Data 8
Figure S1: Example one-colour plots of hydrolysis assays for pentaplex reference gene panel targets 8
Figure S2: Example one-colour plots of Rainbow™ assays for reference gene panel targets. 9
Figure S3: Example two-colour plots of hydrolysis assays for reference gene panel targets 10
Figure S4: Example two-colour plots of Rainbow™ assays for reference gene panel targets 11
Figure S5: DNA copy number concentration comparison between reference genes tested in uniplex and pentaplex. 12
Table S9: Average copies per partition (ʎ) of gBlock™ reference gene mix 13
Table S10: Average Copies per partition (ʎ) hgDNA 13
Table S11: Average copies per partition (ʎ) of cfDNA extracts 13
Figure S6: gBlock™ reference gene mix linear regression per reference gene 14
Table S12: gBlock™ reference gene mix linear regression equation per reference gene for hydrolysis and Rainbow™ assay chemistries 15
Figure S7: hgDNA linear regression plots per reference gene 16
Figure S8: hgDNA pairwise differences in log concentration observed between the genes. 17
Table S13: Standard deviation (%CV) of hgDNA dilution series per reference gene. 18
Table S14: Relative standard deviations expressed as percentages based on mean of all five reference genes. 18
Table S15: Standard deviation (%CV) of cfDNA extracts per reference gene 18
Figure S9: Platform comparison assessment on hgDNA and cfDNA. 20
Figure S10: hgDNA fragment profile assessment by automated gel-electrophoresis system TapeStation 21

Section 1: Supplementary Methods

Table S1: Restriction enzyme digestion protocol

Components	Stock	Final	Unit	Reaction volumes (µL)
HindIII	20	1	U/µL	2.5
NEBuffer™ r2.1 Buffer	10	1	X	5
hgDNA	100	1	µg	5
Nuclease-free Water	-	-	-	37.5
Total				50

Reaction mix was incubated at 37°C for 1 hour followed by heat inactivation at 80°C for 20 minutes.

Table S2: gBlock™ information

Gene Symbol / gBlock Name	Accession number (location)	Amplicon Length (bp)	gBlock length (bp)
RPPH1	NC_000014.8 (545,984-546,113)	64	130
RPS27A	NC_000002.12 (55,235,155-55,235,284)	97	130
HBB	NC_000011.10 (5,227,143-5,227,272)	76	130
PMM1	NC_000022.10 (41,577,650-41,577,779)	78	130
DCK	NC_000004.12 (71,022,367-71,022,496)	83	130

Each of the gBlock™ were prepared according to the manufacturer's instructions and diluted to ~5000 copies/µL based on manufacturer quantification estimate using normalisation metric based on optical density. dPCR copy number measurement was performed per reference gene with matched gBlock™. A mixture of gBlock™ sample was prepared with ~5000 copies/µL of reference gene gBlock™ at 1:1 ratio.

Table S3: Hydrolysis Assay Oligonucleotide information

Gene Symbol / Assay Name	**Accession number / Genome location**	**Oligo Name**	**Sequence (5'->3')**	**Amplicon length (bp)**	**5' Hydrolysis Probe fluorophore / 3' quencher**	**Design**
RPPH1	NC_000014.8<br>Chr 14q11.2	RPPH1_Fwd<br>RPPH1_Rev<br>RPPH1_Prb	F:GCGGAGGGAAGCTCATCAG<br>R:GGACATGGGAGTGGAGTGACA<br>P:CACGAGCTGAGTGCG	64	HEX / MGBEQ	[1]
RPS27A	NC_000002.12<br>Chr 2 p16	RPS27A_Fwd<br>RPS27A_Rev<br>RPS27A_Prb	F:cgggtttgggttcaggtctt<br>R:tgctacaatgaaaacattcagaagtct<br>P:tttgtctaccacttgcaaagctggccttt	97	ATTO 550 / BHQ-2	[2]
HBB1	NC_000011.10<br>Chr 11 p15.5	HBB_Fwd<br>HBB_Rev<br>HBB_Prb	F:gctgagggtttgaagtccaactc<br>R:ggtctaagtgatgacagccgtacct<br>P:agccagtgccagaagagccaagga	76	FAM / BHQ-1	
PMM1	NC_000022.10<br>Chr 22 q13.2	PMM1_Fwd<br>PMM1_Rev<br>PMM1_Prb	F:cccctaagaggtctgttgtgttg<br>R:aggtctggtggcttctccaat<br>P:caaatcacctgaggtcaaggccagaaca	78	Cy5 / BHQ-2	
DCK	NC_000004.12<br>Chr 4 q13.3	DCK_Fwd<br>DCK_Rev<br>DCK_Prb	F:tggtgggaatgttcttcagatga<br>R:tcgactgagacaggcatatgtt<br>P:tgtatgagaaacctgaacgatggt	83	ROX / BHQ-2	
HER2	NC_000017.11<br>Chr 17 q12	HER2_Fwd<br>HER2_Rev<br>HER2_Prb	F: TGCACTTCCCAGGATTAGGG<br>R: CAGGTAGCAGGGGGAGATGT<br>P: AAAGACCGGGTAGGGTCTGTCTCC	71	FAM / BHQ-1	[3]

Manufacturer: LGC BioSearch, Dual HPLC Purified.

Table S4: Pxlence Rainbow™ Assay Oligonucleotide information

Gene Symbol / Assay Name	Accession number / Genome Location	Oligo Name	Sequence (5'->3')	Amplicon length (bp)	Universal Probe fluorophore / Probe description	Design
RPPH1	NC_000014.8 Chr 14q11.2	RPPH1_Fwd (Rainbow™)	F:GCGGAGGGAAGCTCATCAG	64	HEX / RBO-01-C-HEX	[1]
		RPPH1_Rev	R:GGACATGGGAGTGGAGTGACA			
RPS27A	NC_000002.12 Chr 2 p16	RPS27A_Fwd (Rainbow™)	F:cgggtttgggttcaggtctt	97	ATTO 550 / RBO-01-A-550	[2]
		RPS27A_Rev	R:tgctacaatgaaaacattcagaagtct			
HBB	NC_000011.10 Chr 11 p15.5	HBB_Fwd (Rainbow™)	F:gctgagggtttgaagtccaactc	76	FAM / RBO-01-B-FAM	
		HBB_Rev	R:ggtctaagtgatgacagccgtacct			
PMM1	NC_000022.10 Chr 22 q13.2	PMM1_Fwd (Rainbow™)	F:cccctaagaggtctgttgtgttg	78	Cy5 / RBO-01-F-CY5	
		PMM1_Rev	R:aggtctggtggcttctccaat			
DCK	NC_000004.12 Chr 4 q13.3-q21.1	DCK_Fwd (Rainbow™)	F:tggtgggaatgttcttcagatga	83	ROX / RBO-01-D-ROX	
		DCK_Rev	R:tcgactgagacaggcatatgtt			

Manufacturer: Pxlence.
Forward primers have been selected as Rainbow™ primer which contains 5' modification to allow the Rainbow™ probe to bind. Reverse primers are regular primers.

Table S5: Assay preparation

	Hydrolysis			*Pxlence Rainbow™*			
ID	**Oligonucleotide concentration in dPCR reaction, µM**	**Reconstituted oligonucleotides stock, µM**	**20X Oligonucleotide mix, µM**	**Oligonucleotide concentration in dPCR reaction, µM**	**Reconstituted oligonucleotides stock, µM**	**20X Oligonucleotide mix, µM**	**Total volume (µL)**
Forward	0.9	100	18	0.1	100	2	200
Reverse	0.9	100	18	0.3	100	6	
Probe	0.25	100	5	0.125	7.5	2.5	

The 20X primer-probe mix was prepared using Nuclease-free water. The total volume of primer-probe mix was split into 50 µL aliquots and stored at -20°C. For each dPCR plate, fresh aliquots were used.

Table S6: Reaction Setup

**Components**	**Stock**	**Final**	**Unit**	**Single dPCR reaction mix for Hydrolysis (µL)**	**Single dPCR reaction mix for Rainbow™ (µL)**
Water	-	-	-	1.1	1.1
QIAcuity Probe PCR Kit	4	1	X	3.30	-
One-step Advanced Probe Master Mix	4	1	X	-	3.30
RPPH1	20	1	X	0.66	0.66
RPS27A	20	1	X	0.66	0.66
HBB (HER2*)	20	1	X	0.66	0.66
PMM1	20	1	X	0.66	0.66
DCK	20	1	X	0.66	0.66
Total pre-reaction volume (µL)				7.70	7.70
Template volume (µL)				5.50	5.50
Total dPCR reaction volume (µL)				13.20	13.20

**HER2 assay is only used in HER2 Ratio Panel with hydrolysis assay chemistry only where HBB gene assay is replaced with HER2.*

Table S7: Thermal cycling conditions

Step	Number of cycles	Temp (°C)	Time
Enzyme activation	*1*	*95*	*2 min*
Denaturation	*40 or 50**	*95*	*15*
Annealing/Extension		*60*	*30s*

**40 cycles for hydrolysis, and 50 cycles for Pxlence Rainbow™ assay chemistry cycling conditions were applied.*

Table S8: Imaging Parameters

Channel	Exposure duration (ms)	Gain	Fluorophores used
Green	*500*	*6*	*FAM™*
Yellow	*500*	*6*	*HEX™*
*Orange**	*300 or 400*	*5 or 6*	*ATTO 550*
Red	*300*	*4*	*ROX™*
Crimson	*400*	*8*	*Cy5®*

**For Orange channel, 300 ms of exposure duration with gain of 5 imaging parameters was used for hydrolysis assay chemistry. 400 ms of exposure duration with gain of 6 imaging parameters was used for Pxlence Rainbow™ which was the default imaging parameters set within the software.*

References

1. Devonshire AS, Whale AS, Gutteridge A, Jones G, Cowen S, Foy CA, Huggett JF: **Towards standardisation of cell-free DNA measurement in plasma: controls for extraction efficiency, fragment size bias and quantification**. *Anal Bioanal Chem* 2014, **406**(26):6499–6512.
2. Romsos EL, Kline MC, Duewer DL, Toman B, Farkas N: **Certification of Standard Reference Material® 2372a Human DNA Quantitation Standard**. *NIST Special Publication* 2018.
3. Lianhua Dong XW, Alison Devonshire, Jim Huggett, Steve Ellison, Ana Fernandez Gonzalez, David French, Phattaraporn Morris, Sasithon Temisak, Young Bae, Maxim Vonsky, Andrey Runov, Anna Baoutina, Jacob L.H. McLaughlin, Daniel Burke, Roberto Becht Flatschart, Marcelo N Medeiros, Antonio Marcos Saraiva, John Emerson Leguizamon, Andres Leon Torres, Carla Divieto, Mattia Pegoraro, Laura Revel, Burhanettin Yalcinkaya, Hava Taslak, Muslum Akgoz: **CCQM-K176 Breast cancer biomarker HER2 copy number variation (CNV) measurement**. In., vol. 61. Metrologia; 2024.

Section 2: Supplementary Data

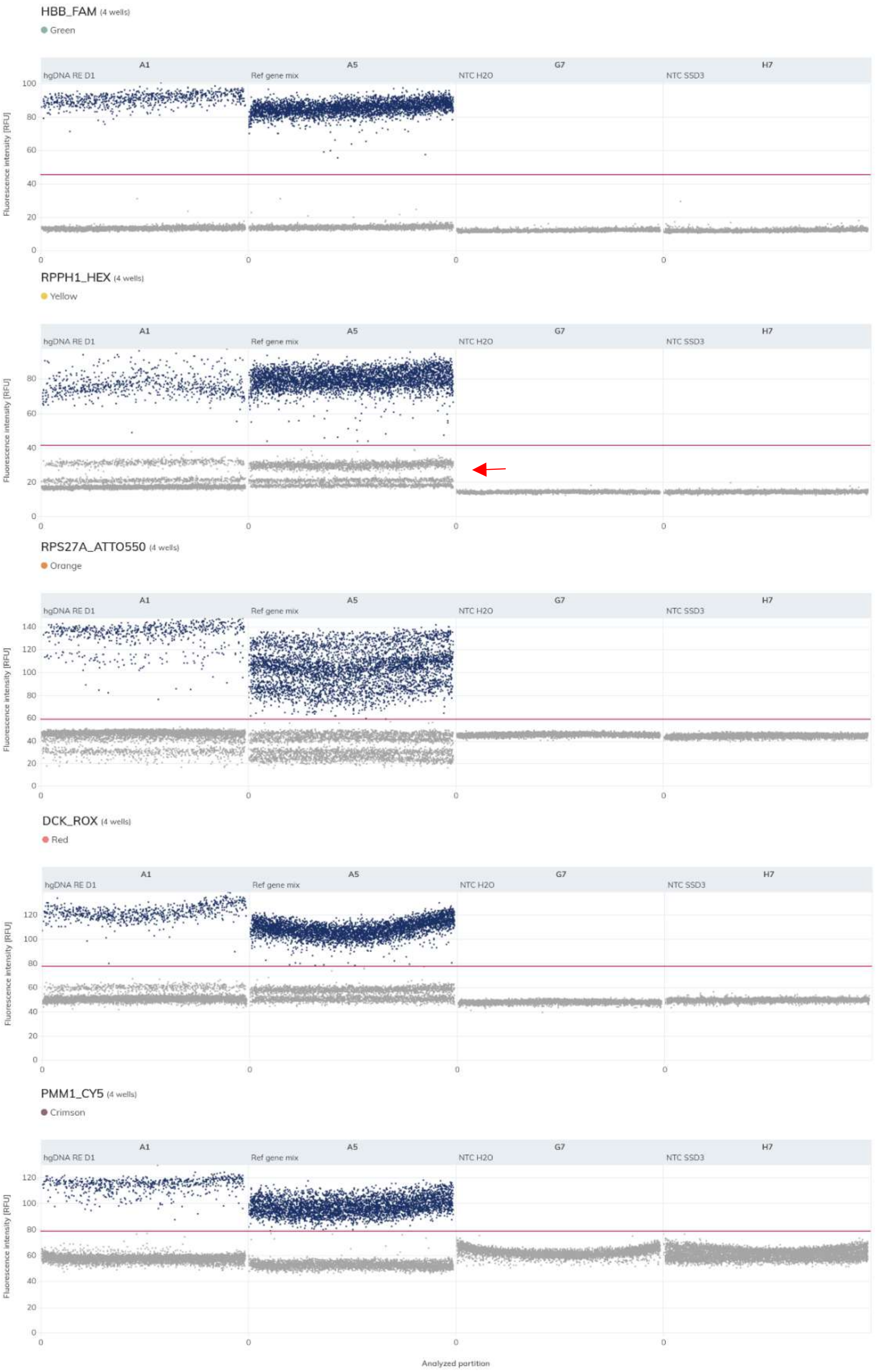

Figure S1: Example one-colour plots of hydrolysis assays for pentaplex reference gene panel targets. One-colour plots of Hydrolysis assay demonstrating peak resolution for hgDNA D1 (600 cp/µL) in well A1, gBlock reference gene mix (5000 cp/µL) in well A5, no-template control in well G7, and carrier background in well H7 with HBB, RPPH1, RPS27A, DCK and PMM1 reference gene targets (top to bottom order) multiplexed in all wells.

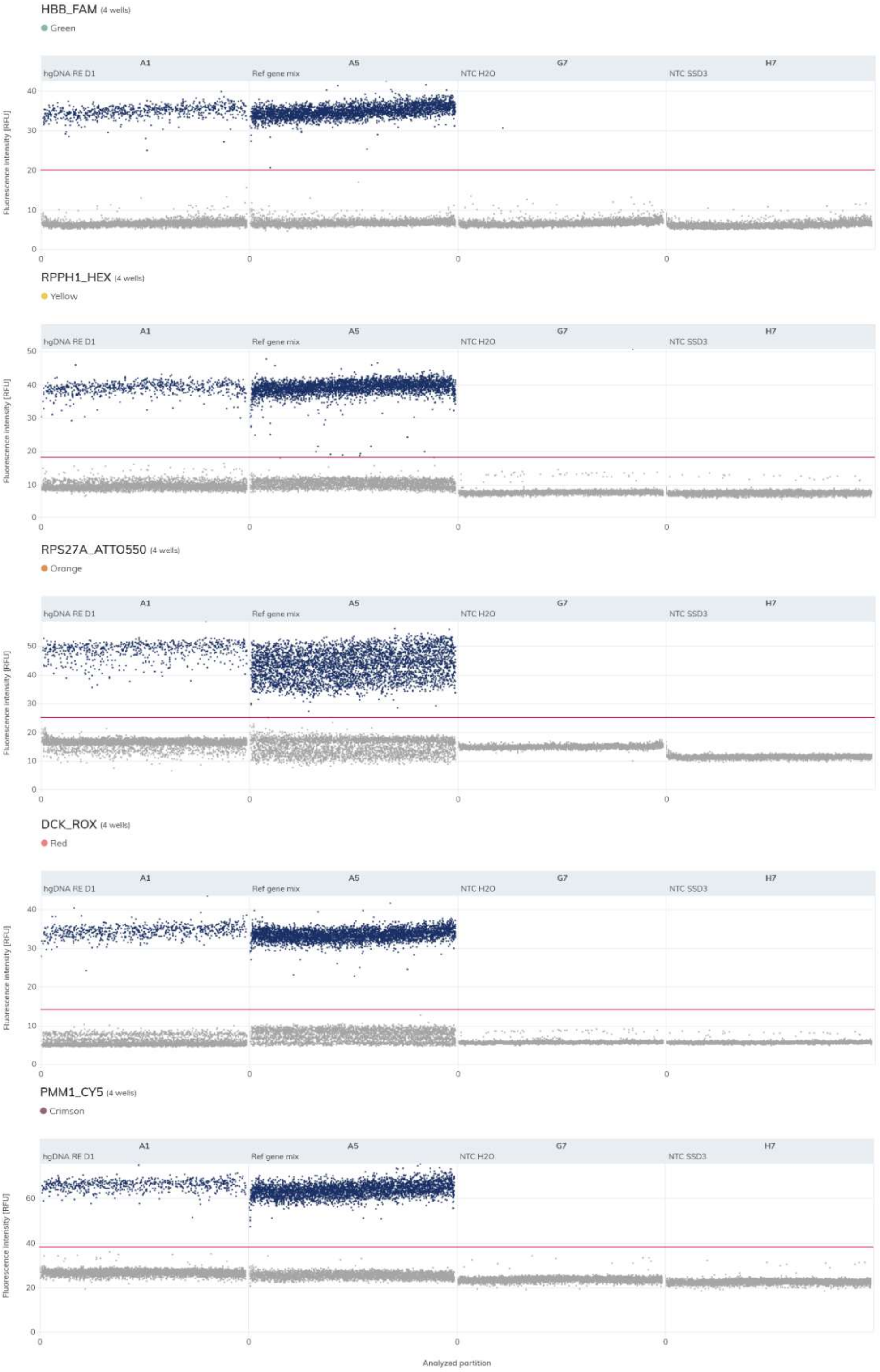

Figure S2: Example one-colour plots of Rainbow™ assays for reference gene panel targets. One-colour plots of Rainbow™ assay demonstrating peak resolution for hgDNA D1 (600 cp/µL) in well A1, gBlock™ reference gene mix (5000 cp/µL) in well A5, no-template control in well G7, and carrier background in well H7 with HBB, RPPH1, RPS27A, DCK and PMM1 reference gene targets (top to bottom order) multiplexed in all wells.

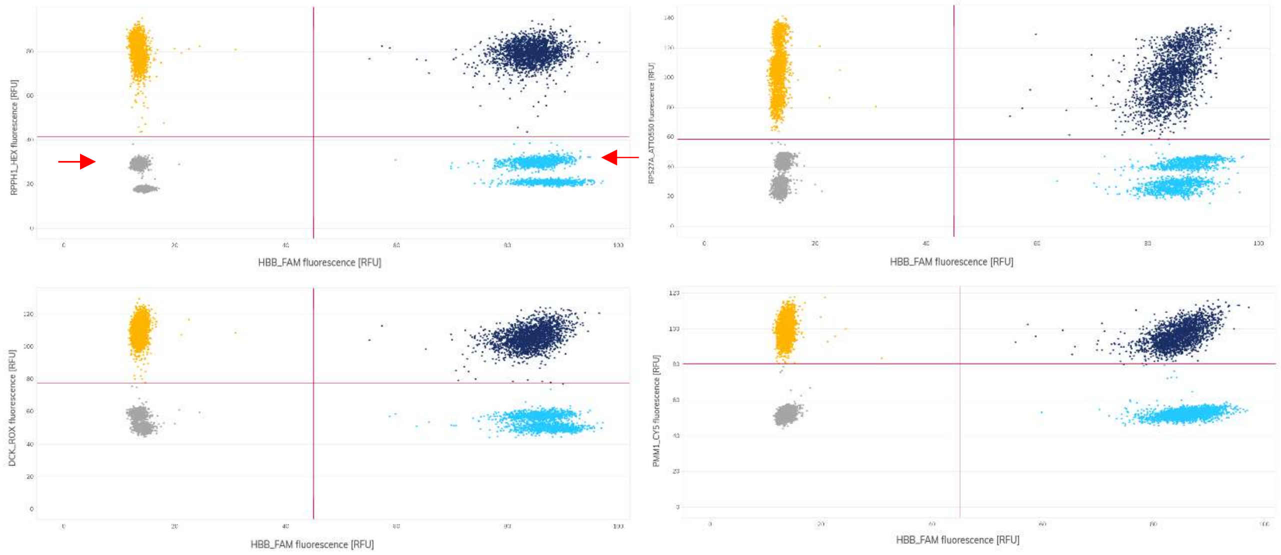

(A)

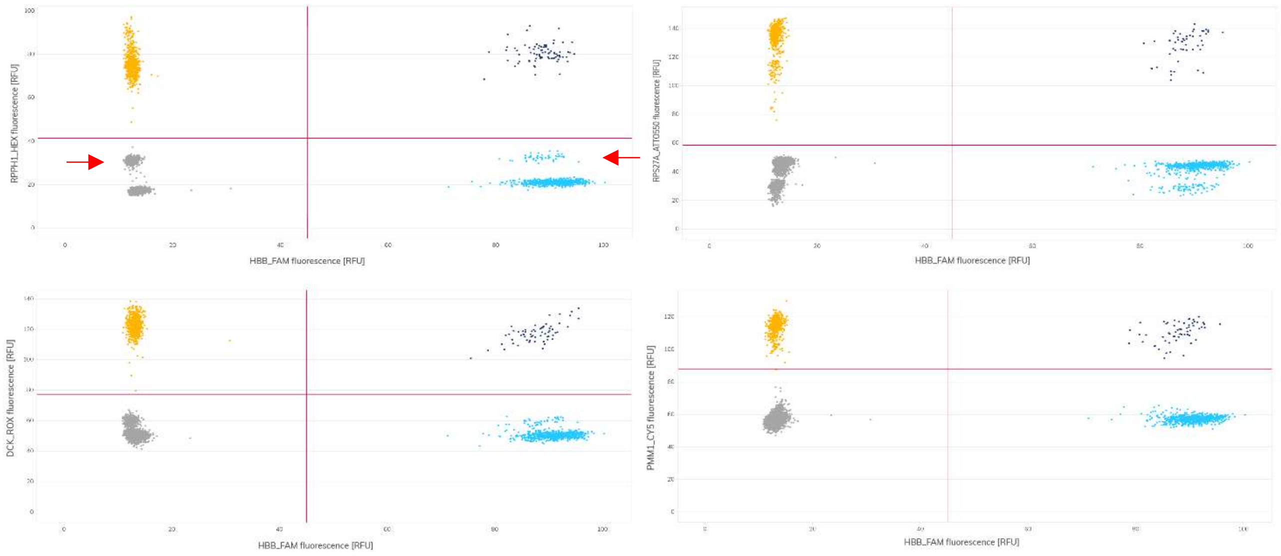

(B)

Figure S3: Example two-colour plots of hydrolysis assays for reference gene panel targets. (A) gBlock^{TM} Reference Gene Mix ~5000 cp/µL with Hydrolysis assay chemistry in well A5. (B) Commercial hgDNA (predigested) ~600 cp/µL with Hydrolysis assay chemistry in well A1.

With a pentaplex assay format, there are minimum of ten two-colour plot combinations available. We have anchored HBB assay on x-axis therefore we provided the full representation of the pentaplex assay format using two colour plots with only four plots. The pentaplex assay format represents 32 possible clusters which contains 8 clusters per quadrant. Each two-colour plot has four quadrants that are separated via threshold containing a cluster of partitions including rain. This influenced the hydrolysis assays in the form of bleed through due to high end-point fluorescence where each of the 8 clusters had suboptimal end-point fluorescence hence multiple clusters appearance per quadrant.

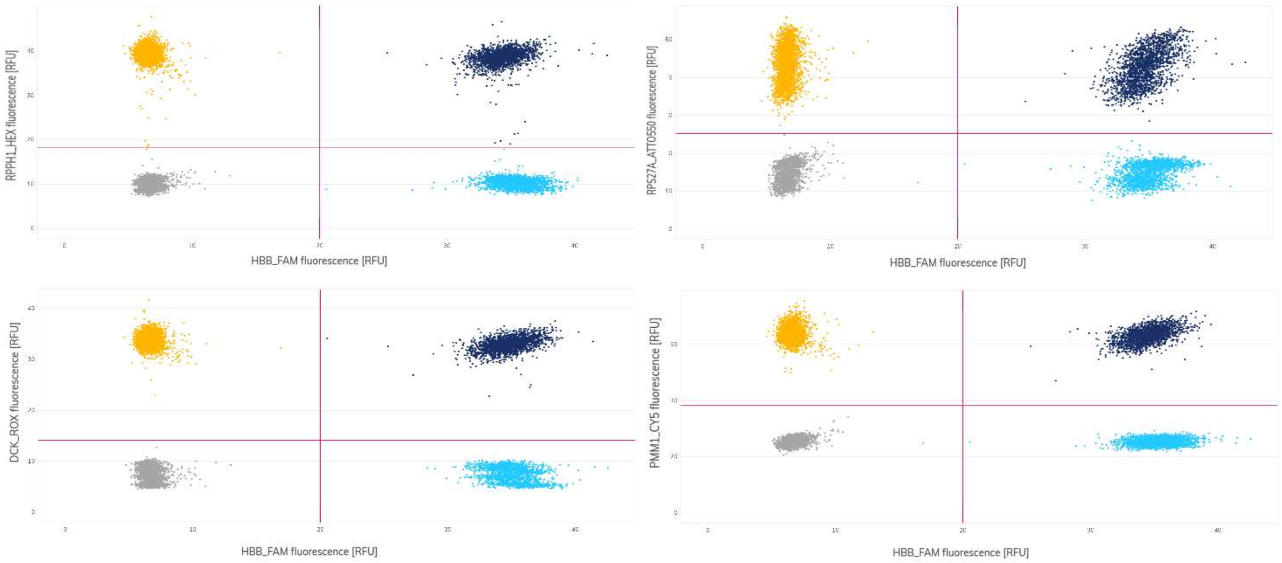

(A)

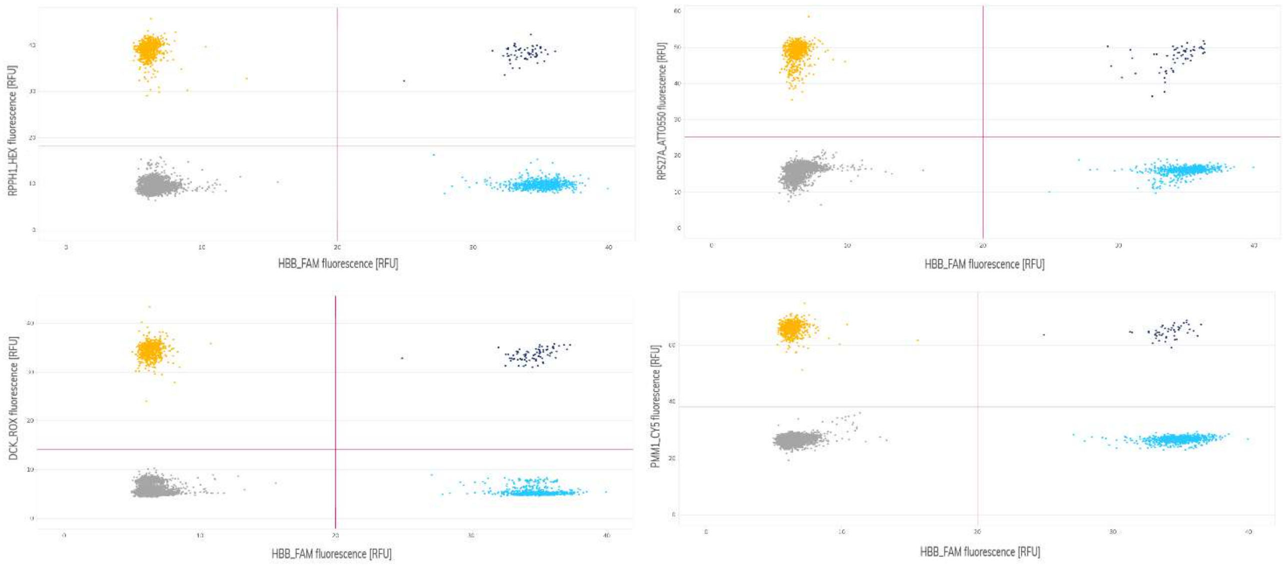

(B)

Figure S4: Example two-colour plots of Rainbow™ assays for reference gene panel targets. (A) gBlock™ Reference Gene Mix ~5000cp/µL with Rainbow™ assay chemistry in well A5. (B) Commercial hgDNA (predigested) ~600 cp/µL with Rainbow™ assay chemistry in well A1.

With a pentaplex assay format, there are minimum of ten two-colour plot combinations available. We have anchored HBB assay on x-axis therefore we provided the full representation of the pentaplex assay format using two colour plots with only four plots. The pentaplex assay format represents 32 possible clusters which contains 8 clusters per quadrant. Each two-colour plot has four quadrants that are separated via threshold containing a cluster of partitions including rain. The influence of bleed-through in the Rainbow™ assays per quadrant was minimal compared to hydrolysis assays.

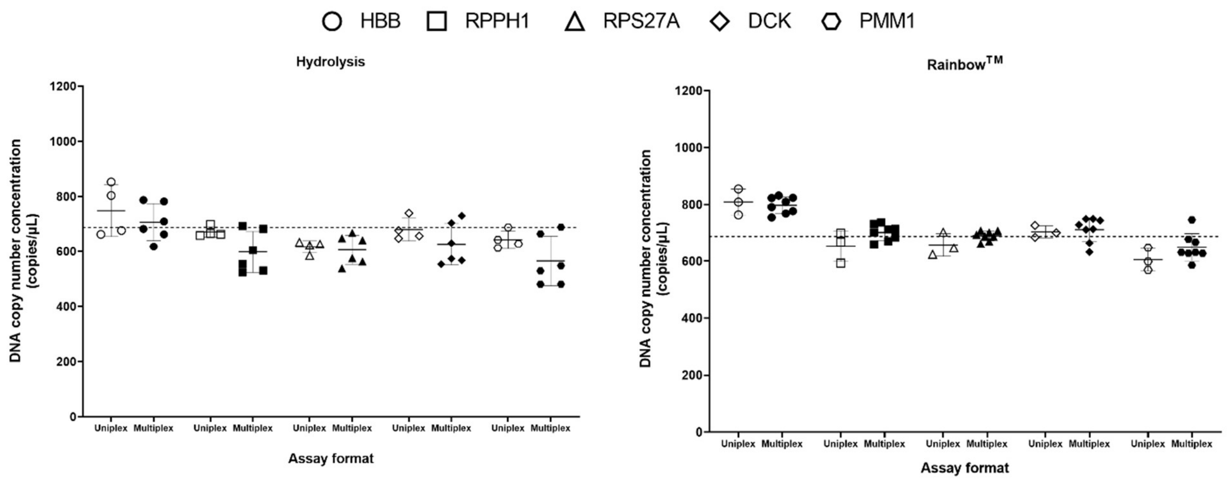

Figure S5: DNA copy number concentration comparison between reference genes tested in uniplex and pentaplex. (A)DNA copy number concentration comparison between reference genes tested in uniplex and pentaplex using hydrolysis assay chemistry. (B) DNA copy number concentration comparison between reference genes tested in uniplex and pentaplex using Rainbow™ assay chemistry.

Both assay chemistries tested separately at a universal melting temperature (60°C) and bespoke cycling conditions (see Table S7). Both uniplex and pentaplex experiments performed separately at different timepoints for each chemistry. For hydrolysis assay chemistry, 4 replicate reactions for uniplex assay format and 6 replicate reactions for pentaplex assay format were performed. For Rainbow™ assay chemistry, 3 replicate reactions for uniplex assay format and 8 replicate reactions for pentaplex assay format were performed. Paired t-test performed between uniplex and pentaplex DNA copy number concentrations per reference gene and no significant differences were observed.

Table S9: Average copies per partition (ʎ) of gBlock™ reference gene mix

	Hydrolysis			Pxlence Rainbow™		
gBlock mix	Average ʎ	SD	CV	Average ʎ	SD	CV
D0	0.70	0.048	6.96%	0.70	0.057	8.11%
D1	0.32	0.021	6.56%	0.32	0.024	7.41%
D2	0.15	0.010	6.75%	0.15	0.010	6.38%
D3	0.073	0.0047	6.42%	0.073	0.0056	7.68%
D4	0.037	0.0031	8.48%	0.036	0.0034	9.46%
D5	0.018	0.0017	9.36%	0.017	0.0017	10.035%
D6	0.0087	0.0010	11.8%	0.0087	0.0012	14.28%
D7	0.0046	0.00083	18.0%	0.0052	0.00085	16.45%
D8	0.0022	0.00052	23.9%	0.0022	0.00051	22.92%

Table S10: Average Copies per partition (ʎ) hgDNA

	Hydrolysis			Pxlence Rainbow™		
hgDNA	Average ʎ	SD	CV	Average ʎ	SD	CV
D1	0.084	0.0096	11.45%	0.085	0.0085	9.98%
D2	0.046	0.0059	13.01%	0.047	0.0040	8.53%
D3	0.023	0.0020	8.46%	0.023	0.0032	14.27%
D4	0.011	0.0011	10.25%	0.011	0.0013	11.63%
D5	0.0053	0.00087	16.47%	0.0054	0.0010	17.80%
D6	0.0028	0.00065	23.61%	0.0027	0.00059	21.64%

Table S11: Average copies per partition (ʎ) of cfDNA extracts

	Hydrolysis		
cfDNA	Average ʎ	SD	CV
extract 1	0.0074	0.0010	12.83%
extract 2	0.0085	0.0014	15.84%
extract 3	0.0094	0.0020	20.98%
extract 4	0.0082	0.0013	15.48%
extract 5	0.0097	0.0021	21.63%
extract 6	0.00808	0.0020	24.79%

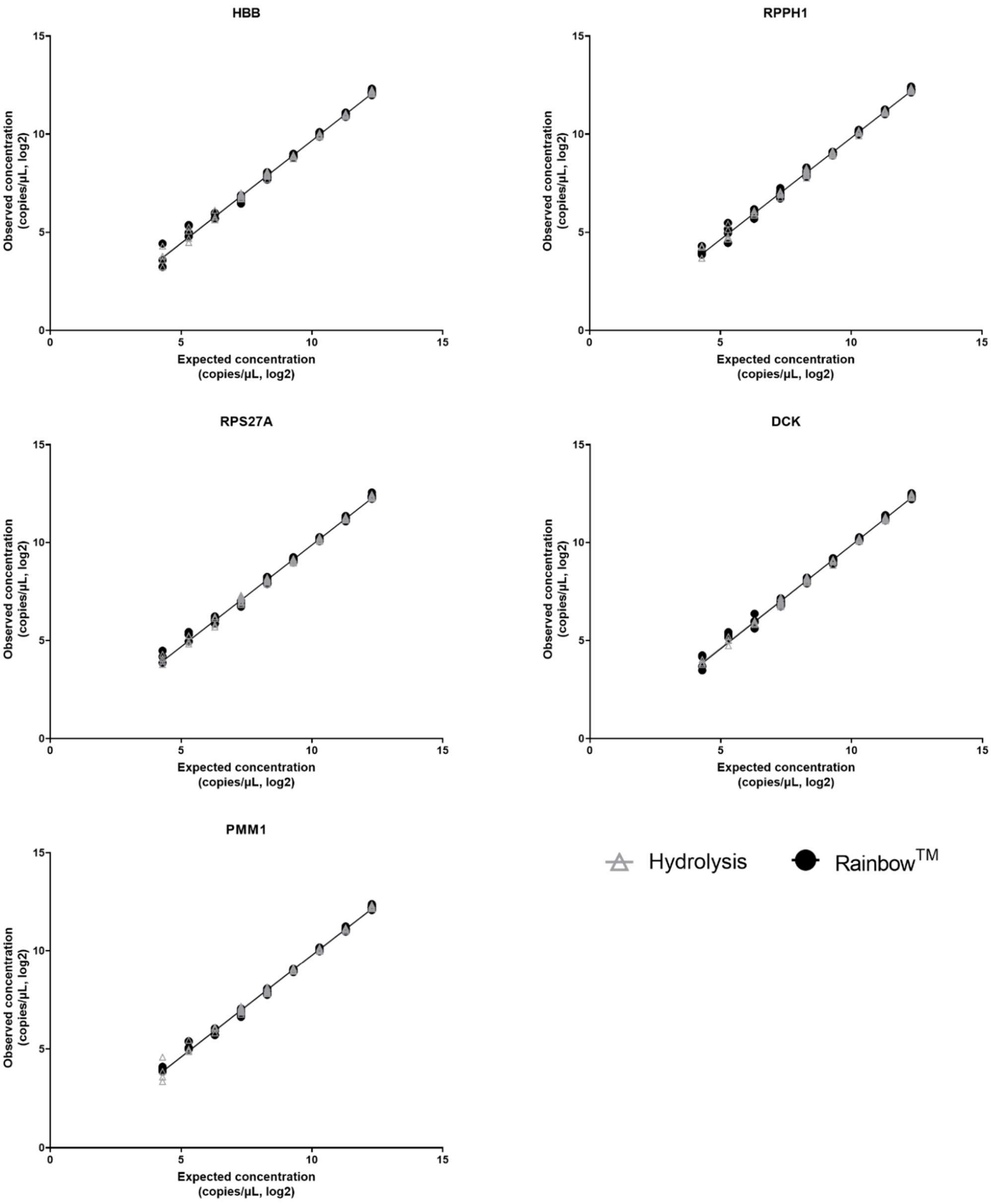

Figure S6: gBlock™ reference gene mix linear regression per reference gene. For the linear regression analysis, two-fold serial dilution was performed yielding nine individual dilutions. For every dPCR run, four replicate measurements were performed per dilution per assay chemistry. The data was log transformed with the base 2 and plotted against the expected log 2 transformed input concentration.

Table S12: gBlock™ reference gene mix linear regression equation per reference gene for hydrolysis and Rainbow™ assay chemistries

	HBB	*RPPH1*	*RPS27A*	*DCK*	*PMM1*
hydrolysis	Y = 1.042*X - 0.7423	Y = 1.038*X - 0.5619	Y = 1.040*X - 0.5352	Y = 1.056*X - 0.6999	Y = 1.034*X - 0.5693
Rainbow™	Y = 1.046*X - 0.7933	Y = 1.038*X - 0.5616	Y = 1.038*X - 0.5029	Y = 1.049*X - 0.6152	Y = 1.036*X - 0.6070

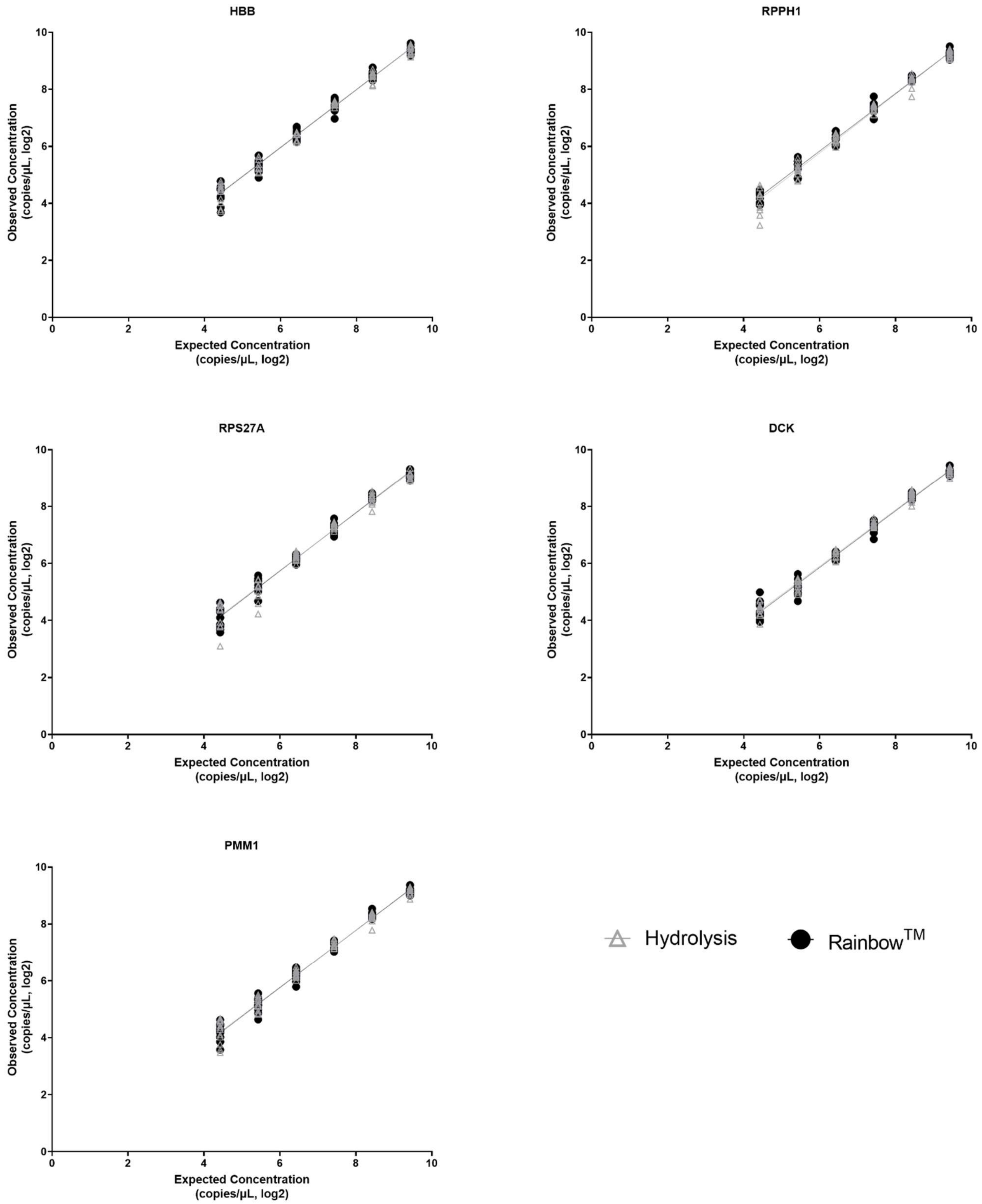

Figure S7: hgDNA linear regression plots per reference gene. For the linear regression analysis, two-fold serial dilution was performed yielding six individual dilutions. For every dPCR run, four replicate measurements were performed per dilution per assay chemistry. The data was log transformed with the base 2 and plotted against the expected log 2 transformed input concentration.

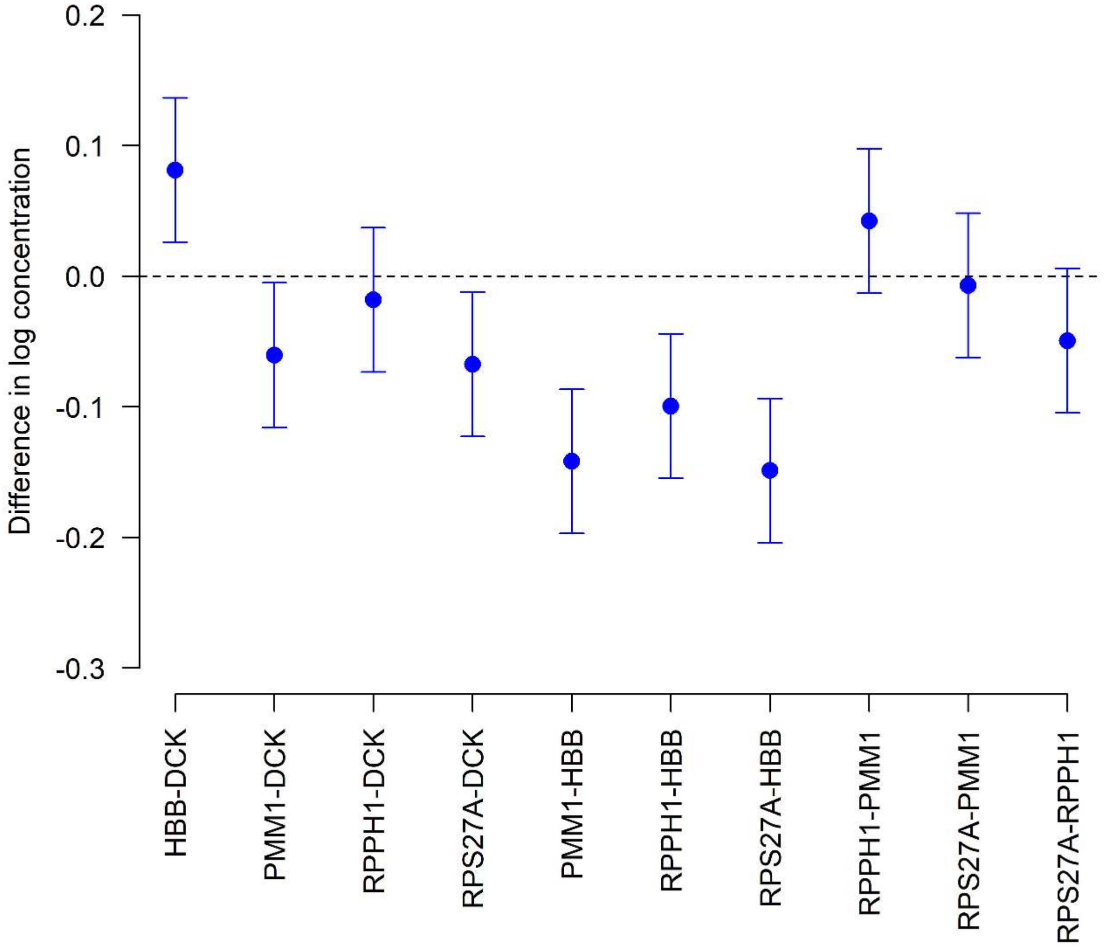

Figure S8: hgDNA pairwise differences in log concentration observed between the genes. Error bars represent 95% confidence intervals based on 2 degrees of freedom.

Table S13: Standard deviation (%CV) of hgDNA dilution series per reference gene.

	HBB		*RPPH1*		*RPS27A*		*DCK*		*PMM1*	
	Mean	CV	Mean	CV	Mean	CV	Mean	CV	Mean	CV
hgDNA D1	664	9.0%	602	8.8%	550	8.6%	600	8.6%	565	7.3%
hgDNA D2	360	10.0%	323	10.1%	315	9.7%	330	9.3%	308	9.1%
hgDNA D3	179	10.5%	159	11.9%	156	9.7%	162	10.1%	152	9.4%
hgDNA D4	83	10.8%	77	11.5%	73	8.0%	79	8.9%	75	10.9%
hgDNA D5	41	14.6%	38	17.5%	36	20.3%	37	15.3%	36	16.1%
hgDNA D6	21	21.1%	18	20.1%	18	25.7%	21	18.4%	19	23.2%

Table S14: Relative standard deviations expressed as percentages (nlme model) for hgDNA.

		Residual (within-experiment)					
Gene	Experiment	D1	D2	D3	D4	D5	D6
5.91%	2.71%	8.26%	10.04%	9.72%	9.86%	17.55%	23.43%

Table S15: Standard deviations for cfDNA extracts (ANOVA model)

	Mean	*Repeatability* *(S_{within})*	*Between gene* *($S_{between}$)*
cfDNA extract 1	53.46	6.72	N/A
cfDNA extract 2	61.95	8.47	4.77
cfDNA extract 3	57.87	7.34	12.85
cfDNA extract 4	57.44	8.29	3.76
cfDNA extract 5	65.34	11.49	10.20

cfDNA extract 6	55.38	13.49	4.57

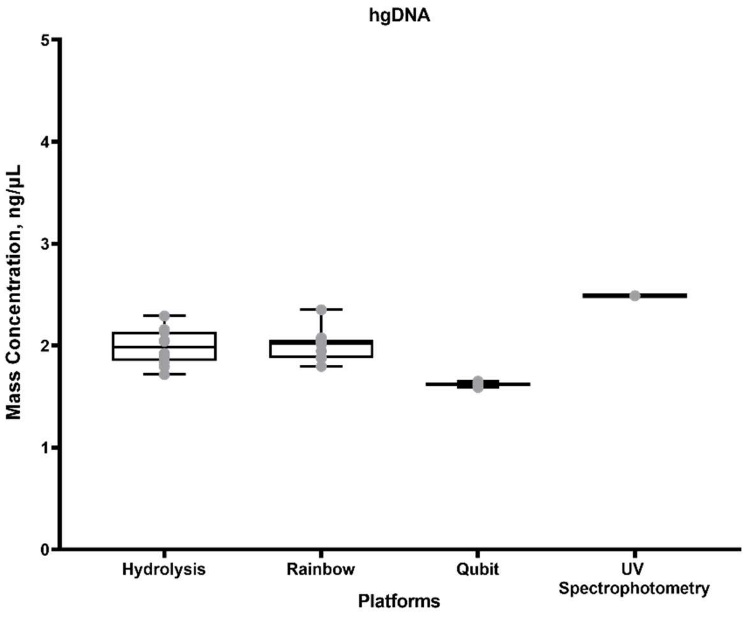

(A)

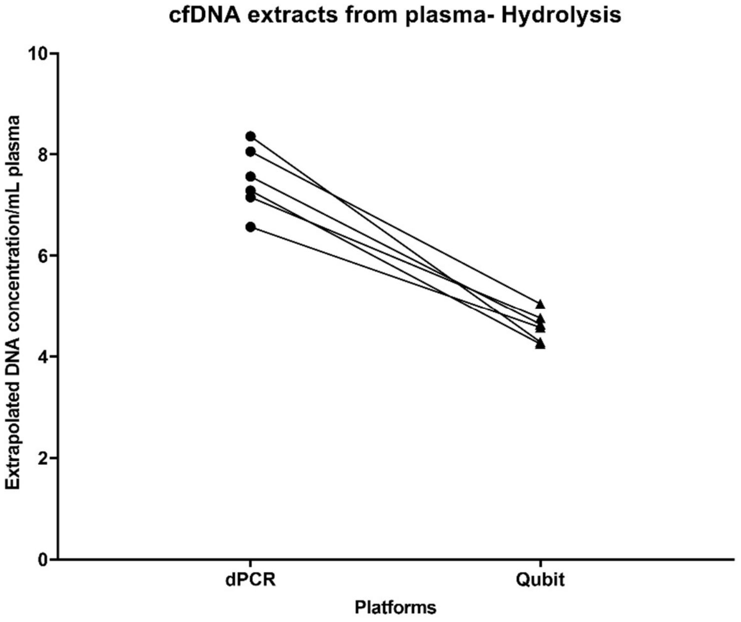

(B)

Figure S9: Platform comparison assessment on hgDNA and cfDNA. (A) hgDNA mass concentration assessment based on 3 platforms: QIAcuity dPCR based copy number measurement including two assay chemistries (hydrolysis and Rainbow^{TM}), Qubit fluorometer (ThermoFisher Scientific, Waltham, Massachusetts, USA) dsDNA broad range assay-based mass measurement, and Manufacturer's value based on UV spectrophotometer mass measurement. QIAcuity dPCR assessment was based on both hydrolysis and rainbow assay chemistry. (B) cfDNA extrapolated DNA concentration assessment based on 2 platforms: QIAcuity dPCR based copy number measurement, and Qubit dsDNA high sensitivity assay-based mass measurement. QIAcuity dPCR assessment was based on hydrolysis assay chemistry only.

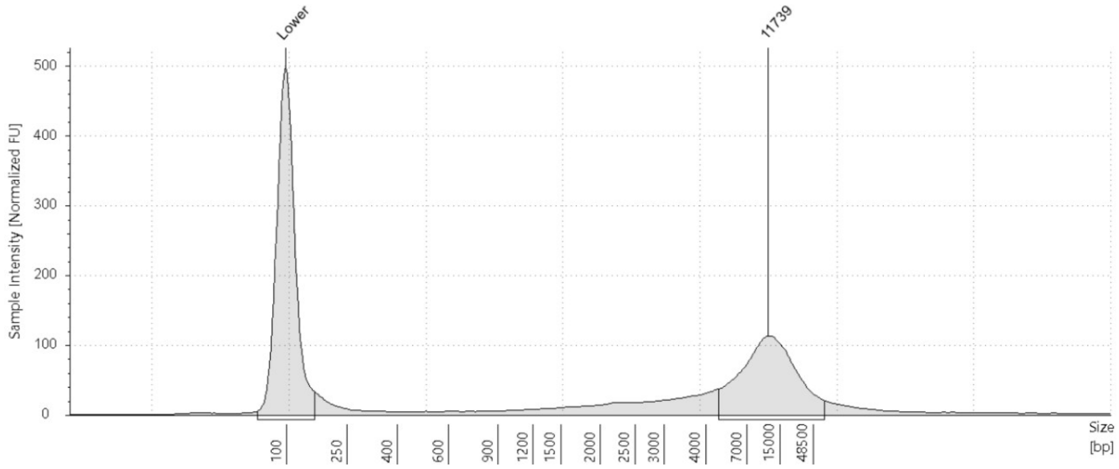

(A)

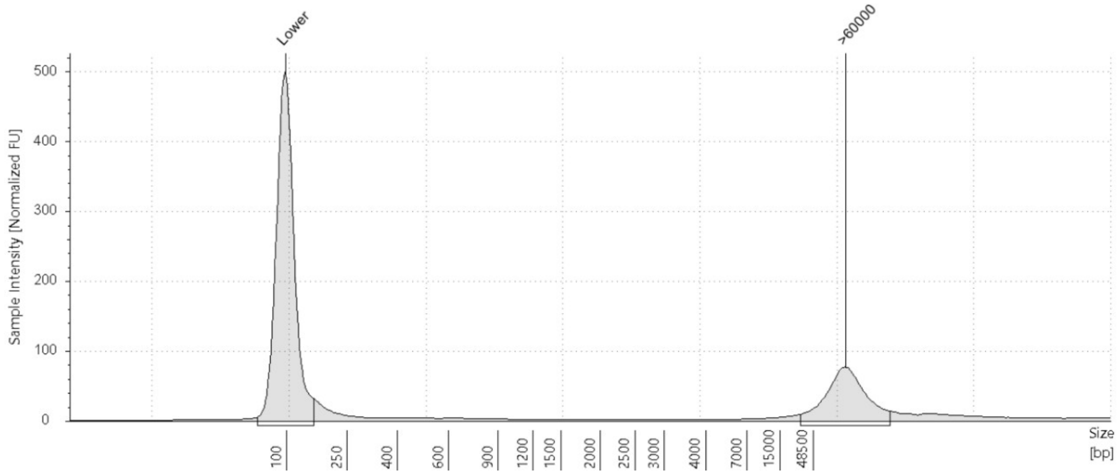

(B)

Figure S10: hgDNA fragment profile assessment by automated gel-electrophoresis system TapeStation. (A) Digested hgDNA fragment profile demonstrating average size of ~11kbp. (B) Non-digested hgDNA fragment profile demonstrating average size of >60kb.
